# Microfluidic Flow-Focusing for Size-Controlled Formation of Cubosomes

**DOI:** 10.1021/acs.langmuir.5c03488

**Published:** 2025-10-17

**Authors:** Celso J. O. Ferreira, Margarida Barros, Marco Fornasier, Alexandre Chícharo, Claudia Botelho, Maria Elisabete C. D. Real Oliveira, Ulf Olsson, Bruno F. B. Silva

**Affiliations:** † 246702 INL-International Iberian Nanotechnology Laboratory, Braga 4715-330, Portugal; ‡ CF-UM-UP Physics Centre, 56059 Minho University, Braga 4710-057, Portugal; § CEB-Centre of Biological Engineering, 450509 Minho University, Braga 4710-057, Portugal; ∥ Division of Physical Chemistry, 5193 Lund University, P.O. Box 124, Lund SE-221 00, Sweden; ⊥ EMPA,Swiss Federal Laboratories for Materials Science and Technology, Center for x-Ray Analytics, Lerchenfeldstrasse 5, St. Gallen 9014, Switzerland; # EMPA,Swiss Federal Laboratories for Materials Science and Technology, Laboratory for Biointerfaces, Lerchenfeldstrasse 5, St. Gallen 9014, Switzerland; ∇ EMPA,Swiss Federal Laboratories for Materials Science and Technology, Laboratory for Biomimetic Membranes and Textiles, Lerchenfeldstrasse 5, St. Gallen 9014, Switzerland

## Abstract

Cubosomes—colloidal dispersions of lipid inverted bicontinuous cubic phases—are promising nanosystems for advanced drug delivery applications. Here, we report on a microfluidic hydrodynamic flow-focusing approach for solvent exchange that enables the preparation of cubosomes with tunable size. The approach involves a preliminary step where the lipid phytantriol, the main building block, is first dissolved in ethanol to create a precursor solution. This precursor is subsequently flowed through the central channel of a cross-shaped microfluidic device, where it is focused by two lateral streams of water containing the stabilizer Pluronic F127. As water and ethanol mix, the polarity shift forces phytantriol to self-assemble into cubosomes, while the laminar-flow conditions imposed by the microchannels provide controlled mixing conditions. By adjusting the flow rate ratio (*Q*_*R*_) between the water-F127 side streams and the phytantriol-ethanol precursor, we control the width of the precursor stream—the region through which solvent molecules exchange. This manipulation thus directly governs the mixing time, influencing the kinetics of lipid self-assembly, which ultimately determines particle size. This technique allows for the tuning of cubosome sizes from 195 nm down to 125 nm, with size decreasing monotonically as *Q_R_* increases and the polydispersity index remaining in the low-to-moderate range. Although variability is still significant, largely due to pumping instability and the use of a commercial microfluidic device not specifically designed for this application, the trends are statistically significant for every precursor-concentration series (*p* ≤ 0.011). Further customization of the apparatus is expected to improve reproducibility and scalability. The results obtained with this microfluidic method differ markedly from those obtained by bulk solvent exchange, which shows only a weak and generally nonsignificant trend toward larger particle size at higher dilution ratios.

## Graphical abstract

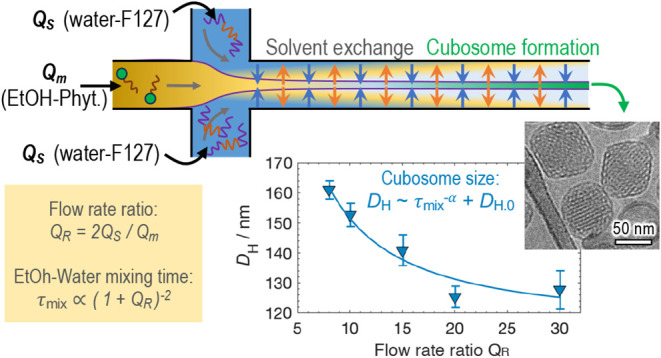

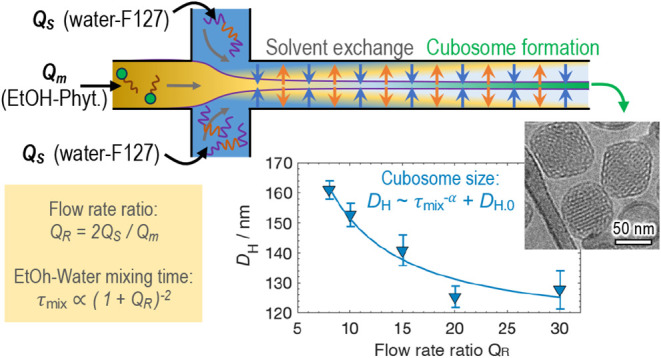

## Introduction

Cubosomes, first introduced by Kåre Larsson, are nanostructured colloidal dispersions of lipid bicontinuous cubic phases, typically stabilized by amphiphilic block copolymers such as F127 and F108, among others.^1–6^ These dispersions consist of a three-dimensional lipid bilayer network enveloping two interpenetrating water channel networks arranged in a lattice of cubic symmetry.^7,8^ The presence of both hydrophobic and aqueous domains make these particles suitable for encapsulating both polar and nonpolar molecules, a feature of interest to many pharmaceutical applications.^9–15^

The most common bicontinuous cubic phases linked to cubosomes are the double-diamond phase (*D*) with space group *Pn3̅m*, the gyroid phase (*G*) with space group *Ia3̅d*, and the primitive phase (*P*) with space group *Im3̅m*.^16^ Among these, the *D* phase is particularly prevalent in excess water (where cubosomes are formed) for lipids like monoolein and phytantriol^16–19^ (Figure 1a). However, the incorporation of other lipids and encapsulation of molecules such as siRNA and other drugs can alter the structure to the *G* and *P* phases.^5,20–25^

**Figure 1:**
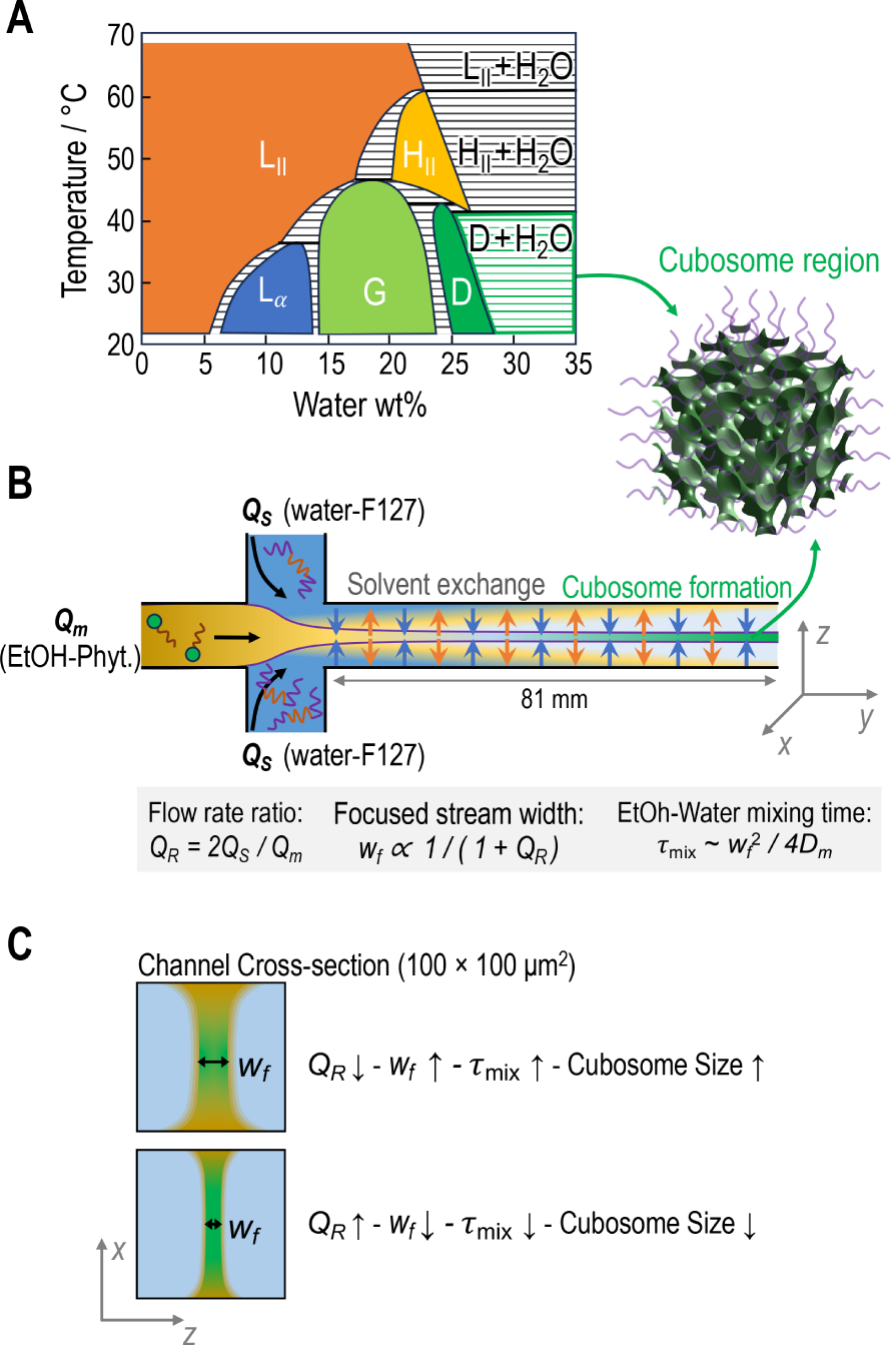
Schematic illustration of the microfluidic solvent-exchange approach for assembling cubosomes with controlled sizes. (A) Phytantriol-water phase diagram (redrawn from Barauskas and Landh^18^), highlighting the two phase region (D+H_2_O) where cubic diamond (D) and excess water coexist, and where cubosomes are formed at high dilutions. (B) Microfluidic approach, where the ethanol-phytantriol solution, with flow rate *Q*_*m*_, flowing through the central channel is hydrodynamically focused by two lateral 0.1 wt % F127 aqueous solutions, flowing from the side channels, each with a flow rate of *Q*_*S*_. The flow rate ratio *Q*_*R*_ controls the width of the ethanol stream, thereby leading to tunable and controlled solvent exchange to tune cubosome size. (C) Illustration of cross-section of the output channel, showing the narrower focused stream widths (*w*_*f*_) when larger *Q*_*R*_ are used. Narrower *w_f_* leads to faster mixing times (*τ*_*mix*_). These effects are hypothesized to shift the system toward faster-assembly regimes, resulting in smaller cubosome sizes. *D*_*m*_ is the mutual ethanol–water diffusion coefficient.

Despite their potential for advanced drug delivery applications, achieving uniform and tunable cubosome sizes remains challenging,^6,26^ with traditional preparation methods showing inherent limitations in size control. This limitation matters because size strongly influences nanoparticle behavior *in vivo*.^27–30^ In top-down approaches, the parent bicontinuous cubic phases incorporating the therapeutic payload are first assembled and subsequently fragmented into nanosized cubosomes.^31–33^ This process requires high energy input, which may damage sensitive therapeutic cargo. Conversely, bottom-up methods such as solvent-exchange involve a preliminary step where lipids are first dissolved in a water-miscible solvent to form a precursor solution.^34^ Mixing this solution with a stabilizer-containing aqueous phase causes a shift in solvent polarity that reduces lipid solubility, leading to the self-assembly of cubosomes. However, uncontrolled mixing between both solvents at the micro and nanoscales results in limited size control and polydispersity. Given that particle size can strongly influence circulation time, cellular uptake, and therapeutic efficacy in drug and gene delivery,^27–30^ the ability to tailor cubosome size is particularly important.

Microfluidic technologies present a promising solution for controlling the size of self-assembled particles by enabling enhanced manipulation and control of mixing conditions.^35–39^ Jahn et al.^40–42^ demonstrated that combining solvent-exchange^34^ with hydrodynamic flow-focusing^43^ allows for tuning the size of liposomes. In this approach, a lipid-isopropanol precursor solution flows through a central microchannel and is hydrodynamically focused by two aqueous side streams. The flow rate ratio *(Q*_*R*_ *)* between the aqueous side streams and the precursor solution directly influence the resulting liposome size. Higher *Q*_*R*_ values narrow the precursor stream, thus shortening the mixing length (*i.e.*, the length that solvent molecules need to travel to exchange), which results in smaller liposome sizes, until an asymptotic lower limit is reached.^40^ Microfluidic hydrodynamic flow-focusing has also been employed for liposome formation in drug and nucleic acid encapsulation.^41,44–46^

An alternative microfluidic approach to achieve rapid mixing relies on the use of herringbone features on the microchannels, which induce chaotic advection to enhance mixing efficiency.^47^ These devices have been particularly successful for combining lipid nanostructures with nucleic acids,^48–50^ typically producing particles with narrow size distributions. By inducing rapid mixing, this method tends to form limit-size particles, whose size is usually tuned by adjusting the system composition, especially the amount of PEG-lipid stabilizer.^51^

The use of microfluidic devices to enhance control over cubosome formation has centered around herringbone mixers. Kim et al.^23^ demonstrated that using such devices leads to the formation of smaller and more monodisperse cubosomes, with the added ability to encapsulate siRNA for gene silencing applications. Additionally, Hong et al.^52^ and Yu et al.^53^ observed that increasing the total flow rate (*Q*_*T*_) led to a reduction in cubosome size. However, increasing *Q*_*R*_ resulted in either larger particles,^52^ or no clear trend,^53^ contrasting with what would be expected on a hydrodynamic flow-focusing device^40^ and thus highlighting the differences between the two geometries.

In this study, we build on the pioneering work by Jahn et al., who demonstrated how microfluidic hydrodynamic flow-focusing can control liposome self-assembly through tunable mixing.^40^ Drawing on this principle, we apply a similar microfluidic flow-focusing solvent exchange approach to prepare cubosomes with tunable sizes (Figure 1). By dissolving the lipid phytantriol in ethanol and mixing it with an aqueous solution containing the stabilizer F127 in a cross-shaped microfluidic device, we achieve enhanced control over cubosome formation. Our results show that cubosome size can be adjusted by varying the flow rate ratio between the aqueous and precursor streams, with higher *Q_R_* values resulting in smaller particles.

Pilkington et al. have independently reported a related approach using microfluidic hydrodynamic focusing to prepare phytantriol-based cubosomes and hexosomes, with particle size tunable by the flow rate ratio.^54^ Their study, published while the present work was already in progress,^55,56^ broadly corroborates the feasibility of this approach. However, their emphasis lies on compositional diversity (monoolein, phytantriol, and phytantriol/tocopherol acetate), cargo encapsulation, and fusion with model membranes. In contrast the present work provides mechanistic insight into cubosome size as a function of flow and formulation parameters. We show that size decreases systematically with *Q*_*R*_, but is also modulated by initial lipid concentration and by including water in the precursor. This behavior is captured by a simplified Damköhler-number^57^ model, which reflects the interplay between solvent mixing time and lipid self-assembly time. Together, these results provide practical guidelines for tuning cubosome size in the ∼125–195 nm range, directly relevant for drug delivery,^27–30^ using an accessible commercial microfluidic device.

## Experimental Section

### Materials and Equipment

Phytantriol (mixture of isomers) was purchased from TCI (Product No. P1674; >95% purity). Ethanol (EtOH) was purchased from Merck (Product No. 1.02371, SupraSolv, for gas chromatography; > 99.8% purity). The stabilizer, Pluronic F127, was purchased from Merck (Product No. P2443). Milli-Q water was used in all experiments.

The experiments were conducted using a cross-shaped microfluidic device made of Cyclic Olefin Copolymer (COC) with 100 × 100 μm^2^ (width × height) cross-section, purchased from Microfluidic ChipShop (catalog no. 02-0757-0166-02). The main (outlet) channel, where mixing occurs, has a length of 81 mm. Attempts to use fabricated polydimethylsiloxane (PDMS) devices were not successful. Despite applying established protocols to extract soluble components from cross-linked PDMS (using diisopropylamine, ethyl acetate, and acetone),^58^ the ethanol in the precursor solution continued to extract impurities from the PDMS matrix, which precipitated upon contact with the aqueous F127 solution,^55^ leading to contamination during cubosome formation.

Fluid flow was controlled using two syringe pumps from New Era Syringe Pumps, models NE-1200 and NE-300. Teflon tubing from IDEX Health and Science, part number 1548, was used to connect the syringes to the microfluidic device.

Hamilton Gastight glass syringes connected to Agani needles were used to inject the solutions into the microfluidic device. The side fluids were injected using 500 μL syringes (model 750LT SYR), and the precursor solution was injected with 25 μL syringes (model 702 SYR).

## Methods

### Solvent Exchange in Microfluidics

In this work, we employ a microfluidic hydrodynamic flow-focusing approach to achieve solvent exchange, under controlled conditions, of an ethanolic solution of phytantriol with an aqueous solution containing 0.1 wt % F127 stabilizer. We use a cross-shaped microfluidic device with 100 × 100 μm^2^ square channels and an 81 mm-long outlet channel where mixing takes place. The precursor phytantriol-ethanol solution, with phytantriol concentration *C*_*phyt.init*_, is introduced through the middle inlet at a flow rate *Q*_*m*_ and is hydrodynamically focused by two side streams of F127 aqueous solution, each entering the device through the side inlets at a flow rate *Q*_*s*_. Under this setup, depicted in Figure 1b, flow is governed by the total flow rate *Q*_*T*_ and flow rate ratio *Q*_*R*_, defined as

1 $$Q_{T}=Q_{m}+2Q_{S}$$

2 $$Q_{R}=2Q_{S}/Q_{m}$$

As the middle and side streams meet at the channel cross, the phytantriol stream is hydrodynamically focused, significantly narrowing its width. If, as a simplification, the viscosity difference between the ethanolic and aqueous solutions is neglected, the height-averaged width of the focused stream *w̅*_*f*_ can be approximated by

$${\mathrm{w̅}}_{f}∼w_{c}/\left[1.5\cdot \left(1+Q_{R}\right)\right]$$ 3

where *wc* is the width of the microchannel, and the factor of 1.5 accounts for the fact that the speed of the focused sheet of fluid is ca. 1.5 times faster than the average flow velocity. This relationship indicates that *w̅*_*f*_ decreases as *Q*_*R*_ increases, with the rate of decrease becoming progressively smaller at higher *Q*_*R*_. Confining the ethanolic flow to a narrower central stream reduces the diffusion path *w*_*f*_ for solvent exchange and thus shortens the time required (*τ*_*mix*_) for mixing. *τ*_*mix*_ can be approximated by

4 $$\tau_{mix}∼w_{f}^{2}/\left(4D_{m}\right)$$

where *D*_*m*_ is the mutual diffusion coefficient of water and ethanol (note that *D*_*m*_ varies with the relative amounts of the two solvents,^59^ but we approximate it here using a constant average value of 6.53 × 10^–10^ m^2^ s^–1^). This variation in *τ*_*mix*_ across different *Q*_*R*_ values is expected to influence the balance between mixing and assembly time scales, thus influencing cubosome size. It should be noted that eqs 3 and 4 provide only a first-order approximation of the mixing time. More accurate estimates could be obtained by directly tracking the mixing of fluorescent dyes or by finite element simulations (*e.g.*, with COMSOL). However, since other relevant time scales governing cubosome formation (*e.g.*, the characteristic lipid assembly time, discussed in Section 3.3) are also not precisely known, this approximation is adequate for the present work.

In this work, *Q*_*T*_ was fixed at 100 μL/min, while *Q*_*R*_ was varied between 8 and 30. This *Q*_*T*_ value represents a compromise: it is high enough to reduce syringe pump pulsation, yet low enough to ensure that the residence time *τ*_*res*_ (defined as the outlet channel length divided by the average fluid velocity) remains significantly greater than *τ*_*mix*_, thus avoiding residence-time effects on particle size.^39^ In this work, *τ*_*res*_ is ∼0.5s and *τ_res_/τ*_*mix*_ ranges from 22 to 270 as *Q*_*R*_ is increased. These conditions ensure complete solvent mixing within the focused stream while keeping the Reynolds number at ∼17, well within the laminar regime. Additionally, since *Q*_*R*_ also acts as a dilution ratio (*Dil*_*R*_), changing the *Q*_*R*_ also changes the concentration of cubosomes:

5 $$Dil_{R}=\frac{V_{F127-aq.}}{V_{lipid-EtOH}}=\frac{2Q_{s}}{Q_{m}}=Q_{R}$$

In eq 5 *V*_*lipid‑EtOH*_ and *V_F127‑aq_* represent the injected volumes of the phytantriol-ethanol precursor solution and the F127 aqueous solution, respectively. To decouple the effect of *Q_R_* on hydrodynamic focusing from its effect on concentration, an additional volume of F127 solution is added at the device outlet to adjust all samples to the same final concentration, corresponding to a *Dil*_*R*_ of 30. This ensures consistent phytantriol and F127 concentration across samples, while also minimizing and standardizing the residual ethanol concentration, which could affect stability through Ostwald ripening. Table S1 details the flow rates, collected volumes, and added F127 volumes for each condition.

### Solvent Exchange in Bulk

For comparison, we also prepared cubosomes through bulk mixing under conditions reproducing the microfluidic process as closely as possible. Before cubosome formation, the F127 solution is kept stirring for 2 min using a magnetic stirring bar. Subsequently, the Phytantriol-ethanol solution is added in a single stroke to meet a dilution ratio equivalent to the flow rate ratio it is being compared to. Thus, in this initial step, the dilution ratios of the cubosome solutions are 8, 10, 15, 20, and 30 (Table S2). These solutions are left stirring for 2 min, after which an aliquot is taken and mixed with an additional volume of F127 solution. This second addition is performed to normalize the concentration to a final *Dil_R_* of 30 for all solutions, with a final volume of 500 μL. As in the microfluidic method, this extra F127 volume ensures that all cubosome preparations, regardless of initial dilution, have the same composition for a more accurate comparison. Table S2 shows the volumes used for each initial *Dil_R_* .

### Small-Angle X-ray Scattering (SAXS)

For structural characterization of the lipid nanoparticles, Small Angle X-ray Scattering (SAXS) was used (Figure 2). Given the dilute nature of the cubosome dispersions (0.032, 0.048, and 0.065 wt % phytantriol for *C*_*phyt.init*_ of 1, 1.5, and 2 wt %, respectively), a concentration step was implemented, by subjecting a considerably large volume of sample to several centrifugation steps using 30,000 NMWL Amicon ultra-0.5 mL Centrifugal Filters (Merck Millipore) until all the volume was filtered. Following this first centrifugation step, water was added to the filter and another centrifugation was carried to remove residual ethanol. A final concentration between 3.9 and 4.4 wt % of phytantriol was achieved.

**Figure 2:**
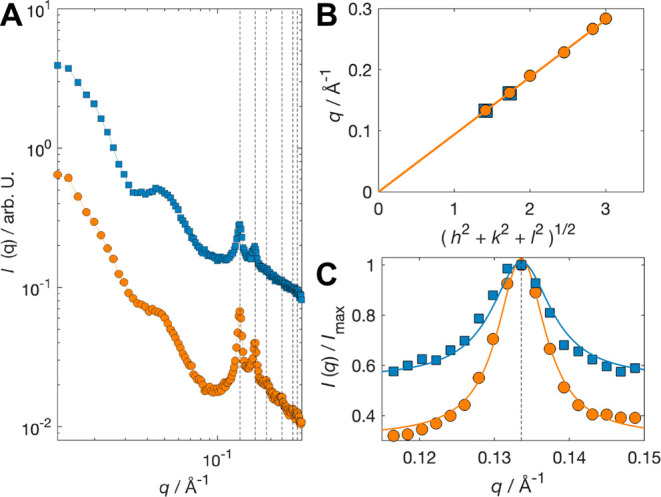
SAXS structural characterization of cubosomes. (A) Scattered intensity from two cubosome samples formed at a Q_R_ of 10 (orange circles) and 15 (blue squares). The sample composition after the concentration step was 4.4 and 3.9 wt % Phytantriol, respectively. The relative peak positions confirm the Pn3̅m space group, with the dashed lines indicating the first six reflections. The data is offset along the vertical axis for ease in visualization. (B) Scattering vector peaks plotted against the square root of the sum of the Miller indexes. By fitting a linear regression line that crosses the origin, one can extrapolate the lattice parameter from the slope following the relation *m* = 2π/a, where a is the lattice parameter of 6.7 nm. (C) Normalized scattering intensity for the main Bragg peak, showing the larger half-width at half-maximum for a Q_R_ of 15. Fitting the peaks with a Lorentzian function (lines) results in domain sizes of *ξ* ∼ 81 and 65 nm for Q_R_ = 10 and 15, respectively.

Measurements were performed using a SAXSLab Ganesha instrument (JJ-Xray, Hoersholm Denmark), equipped with an X-ray microsource (Xenocs, Sassenage, France), with wavelength 1.5406 Å. The generated beam was point collimated and the sample to detector distance was of 480 mm, providing a *q*-range of 0.0125–0.75 Å^–1^. Quartz capillaries of 1 mm diameter were used. Each measurement consisted of 2 h exposure at 25 °C. The scattered photons were collected on a 2D 300 K Pilatus detector (Dectris Ltd., Baden-Daettwil, Switzerland). The isotropic 2D patterns were radially averaged into 1D-scattering patterns, I­(q), where $q=\frac{4\pi }{\lambda }\sin\left\{\theta /2\right\}$ is the magnitude of the scattering vector, θ being the scattering angle, using the instrument software. Calibration of the angular scale was performed using silver behenate as a benchmark.

The Bragg peaks in the SAXS patterns were analyzed to determine the lattice type, structural parameters, and domain size. The scattering vector positions *q*_*hkl*_ for a cubic lattice are given by

6 $$q_{hkl}=\frac{2\pi }{a}\sqrt{h^{2}+k^{2}+l^{2}}$$

where *h*, *k*, and *l* are the Miller indices of the reflections and *a* is the lattice parameter. In bicontinuous cubic phases, the bilayer thickness (δ) can be estimated using the relation:^19,60,61^

7 $$\phi_{phyt}=2A_{0}\left(\delta /\left(2a\right)\right)+\left(4\pi \chi /3\right){\left(\delta /\left(2a\right)\right)}^{3}$$

where *ϕ*_*phyt*_ is the phytantriol volume fraction in the cubic phase, and the constants *A*_0_ = 1.919 and *χ* = −2 apply to the *Pn*3̅*m* space group.

The width of the Bragg peaks provides insights into the domain size (ξ) of the crystal lattice.^62^ To estimate ξ, the first Bragg peak of the cubic phase is fitted using a Lorentzian function of the form

8 $$I\left(q\right)=A/\left(k^{2}+{\left(q-q_{0}\right)}^{2}\right)+B$$

where *A* represents the amplitude, *k* is the half-width at half-maximum (HWHM) of the peak, *q*_0_ is the *q* value of the peak, and *B* is a constant.^63^ Assuming the peak broadening is dominated by finite domain size, the effective domain size *ξ* can then be approximated as ξ≳π/k . Since other factors (*e.g.*, instrumental resolution, thermal fluctuations) also contribute to peak broadening, these values should be regarded as lower bounds. However, because samples have the same composition and were measured under identical conditions, relative comparisons remain valid.

### Cryogenic Transmission Electron Microscopy (Cryo-TEM)

Images of the Phytantriol nanoparticles were captured using a JEM-2200FS transmission electron microscope (JEOL), optimized for Cryo-TEM at Lund University in the National Center for High Resolution Electron Microscopy (nCHREM). Representative data are shown in Figure 3. The JEM-2200FS is equipped with a Schottky field-emission electron source and operated at an acceleration voltage of 200 kV. A cryo pole piece in the objective lens, an in-column energy (omega filter) and a 25 eV filter were also used. Images were recorded via SerialEM software under low-dose conditions onto a bottom-mounted TemCam-F416 camera (TVIPS) operated with 3K by 4K pixels.

**Figure 3:**
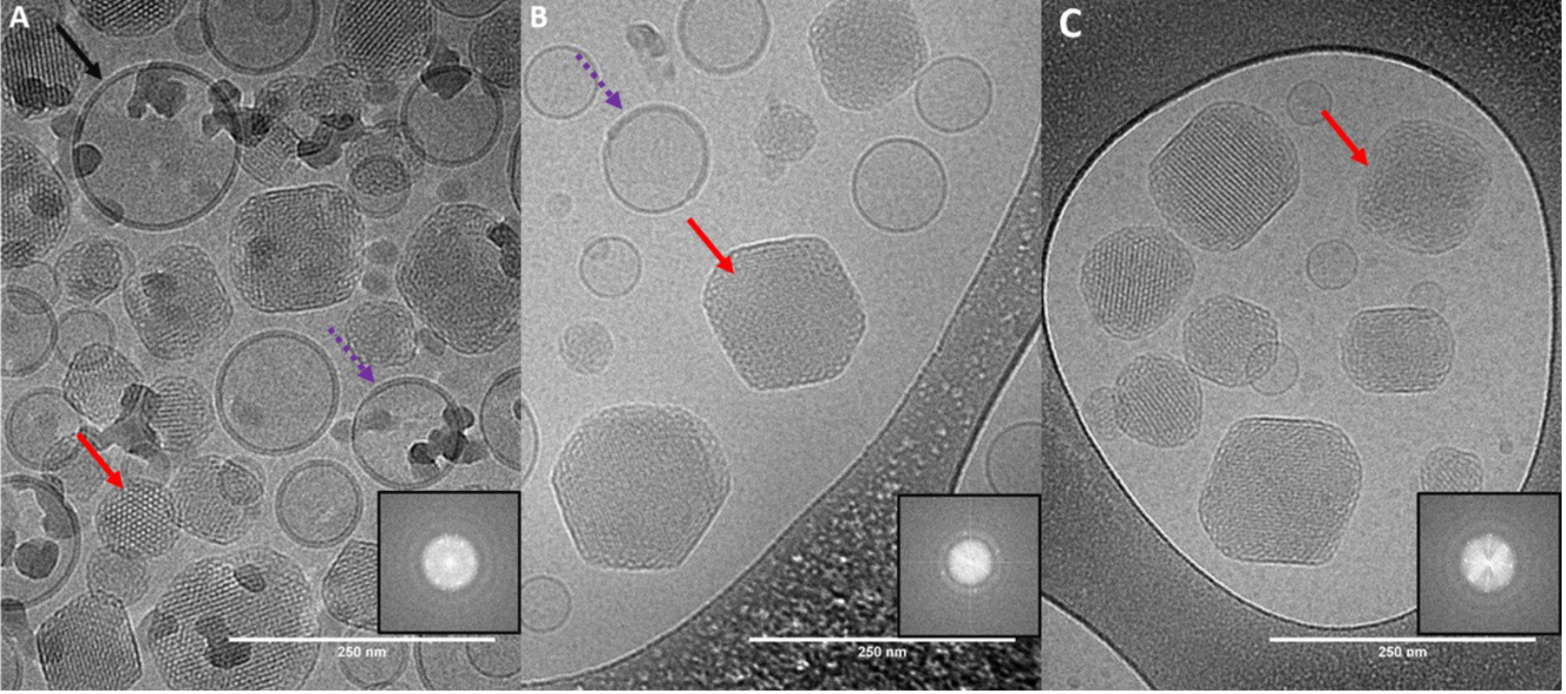
Cryo-TEM images showing the coexistence of cubosomes and vesicles. Panels A, B, and C correspond to samples formed at Q_R_ values of 8, 10, and 15, respectively. Red arrows highlight some of the cubosome particles. Black arrows and purple dashed arrows point to some of the vesicles fully and partially enveloped in the dark diffuse region of ca. 7 nm, respectively. Inserts show 2D Fast Fourier Transforms (FFT) of selected regions (red arrows), highlighting the presence of periodic structures. Scale bars correspond to 250 nm in all images.

Samples were prepared with automatic plunge freezer system (Leica Em GP) with the environmental chamber operated at 20 °C and 90% relative humidity. For each sample a 4 μL droplet was deposited on a lacey Formvar carbon-coated grid (Ted Pella) and was blotted with filter paper to remove extra fluid. The grid was then plunged into the liquid ethane (ca. −184 °C) to ensure the rapid vitrification of the sample in its native state. Before imaging under the microscope, the specimens were then stored in liquid nitrogen (−196 °C) and then transferred using a cryotransfer tomography holder (Fischione Model 2550).

### Dynamic Light Scattering (DLS)

The size of the cubosomes was determined using DLS with the Horiba Nanopartica (SZ-100). For each flow condition, the autocorrelation curves were measured for three independent sample preparations, each measured three times for 60 seconds at two scattering angles, 90° and 173°. To ensure that any potential intermediate structures had enough time to achieve a quiescent cubosome state, the cubosome sizes are always measured by DLS 1 day after cubosome preparation. At the standardized *Dil*_*R*_ of 30, the concentration is dilute enough for cubosomes to diffuse independently. The autocorrelation curves were fitted with a cumulants model described by^64^

$$g^{\left(2\right)}\left(\tau \right)‐1=B+\beta {\left\{exp(‐\overset{¯}{\Gamma }¯\tau )[1+\left(1/2\right)\mu_{2}\tau^{2}]\right\}}^{2}$$ 9

where *B* ≈0 is a baseline term, *β* (close to 1) is the coherence factor, τ is the characteristic mean decay rate, and *μ*_*2*_ is the variance. For freely diffusing particles, *Γ* is given by

10 $$\Gamma =D_{\mathrm{dif.cub}}\cdot q^{2}$$

where *q* is the scattering vector corresponding to the scattering angle, and *D*_*dif.cub*_ is the cubosome diffusion coefficient. By plotting τ as a function of *q* ^*2*^ , *D*_*dif.cub*_ can be obtained from the slope of the resulting linear fit (with the intercept set to zero, see Figure 4b). Finally, by applying the Stokes–Einstein relation, the hydrodynamic diameter *D*_*H*_ of the particles can be calculated from *D*_*dif.cub*_, according to

11 $$D_{H}=\frac{k_{B}\cdot T}{3\cdot \pi \cdot \eta \cdot D_{\mathrm{dif.cub}}}$$

where *k*_*B*_ is the Boltzmann constant, *T* is the absolute temperature, and η is the solvent viscosity.

**Figure 4:**
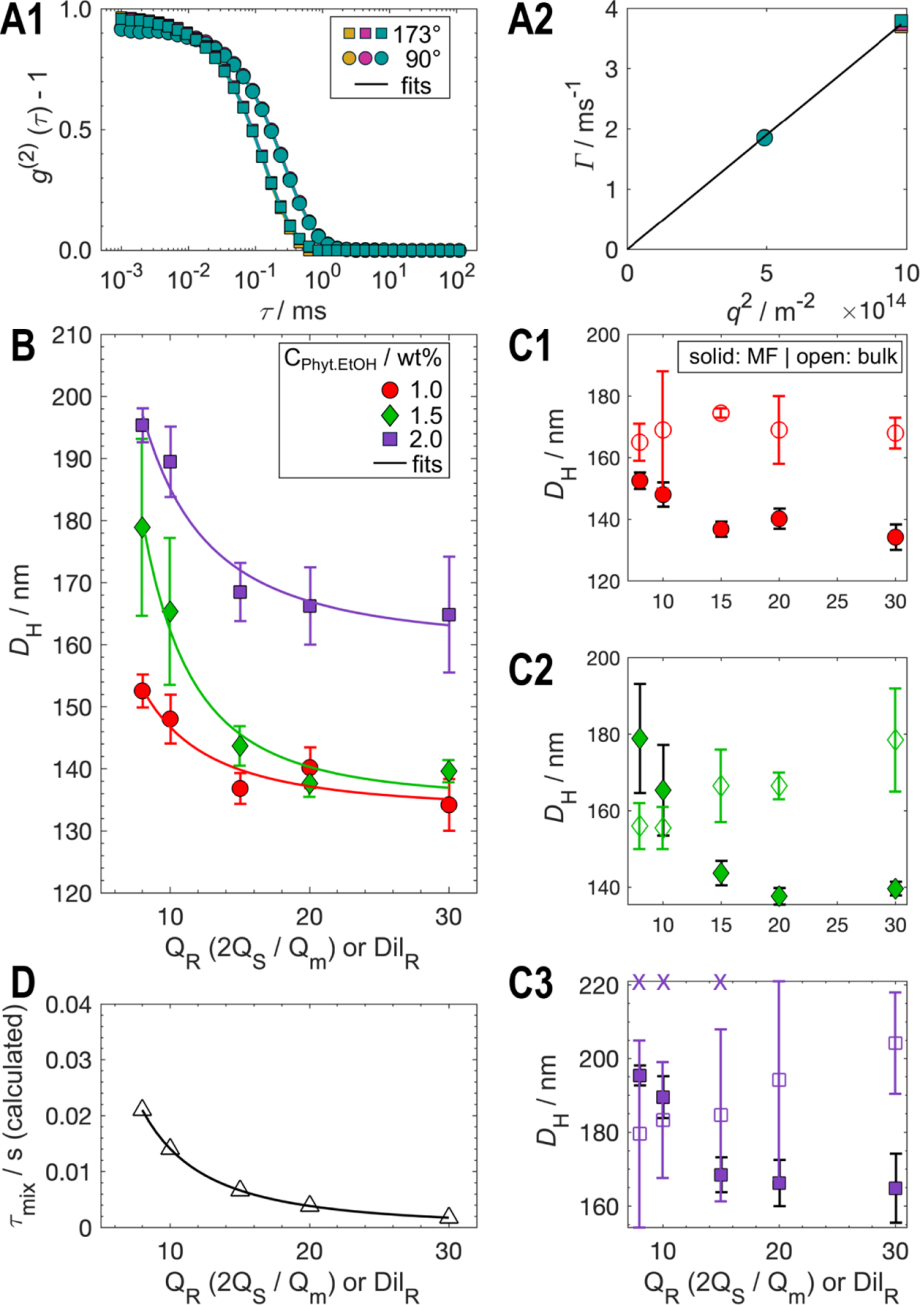
DLS characterization and modeling of cubosome size dependence on flow rate ratio *Q*_*R*_ . (**A**) Representative DLS measurements of cubosomes prepared via microfluidics at *Q*_*R*_ =30 with phytantriol in ethanol concentration (*C*_*phyt.init*_)) of 1 wt %. (A1) Autocorrelation functions g^(2)^(τ)–1 at two scattering angles (90°, circles; 173°, squares), with three repeats per angle. Their near-perfect overlap indicates low polydispersity (PDI = 0.16). (A2) Corresponding decay rates *Γ* vs *q* ^*2*^, used to extract the hydrodynamic diameter *D*_*H*_ (eqs 10–11) from six points (three per angle) plus the intercept. (B) Mean *D*_*H*_ as a function of *Q*_*R*_, for *C_phyt.init_* = 1.0 wt % (red circles), 1.5 wt % (green diamonds), and 2.0 wt % (purple squares). Symbols: experimental values; lines: model fits from eq 15 (cf. Section 3.3). Samples were postdiluted to match final dilution ratio (*Dil*_*R*_) of 30. (C) Comparison of microfluidic (solid) and bulk (open symbols) assembly at equivalent *Dil*_*R*_ (C1–C3). The *D*_*H*_ decrease with increasing *Q*_*R*_ for microfluidics is statistically significant for all concentrations (Table 1), whereas bulk shows only a weak and generally nonsignificant trend toward larger sizes at higher *Dil*_*R*_. Error bars in C and D represent the standard error of the mean. For microfluidics, *N* = 3 for 1.0 and 1.5 wt %, and *N* = 5 for 2.0 wt %; for bulk *N* = 2 for all concentrations. Each *N* = 1 is an independent run. Variability in size is attributed to flow instabilities and the use of a commercial device not specifically designed for this application. Most samples show PDIs in the range 0.15–0.19 (interquartile range), indicating generally narrow distributions. In C3, crosses mark compositions from the second run at *Dil*_*R*_ = 8, 10, and 15, where a secondary micron-sized population was detected. Only the sub-250 nm population is analyzed. (D) Estimated mixing time *τ*_*mix*_ as a function of *Q*_*R*_ (eq 4). Triangles indicate experimental conditions.

The polydispersity index (PDI) is defined as

12 $$PDI=\mu_{2}/\mathrm{\Gamma ̅}$$

Since each scattering angle yields one PDI value, the reported PDI consists of an arithmetic mean of both values. The *D*_*H*_ and PDI values for all conditions, in both microfluidics and bulk experiments, are provided in Tables S3–S6.

### Statistical Analysis

#### Correlation Analysis

We used Kendall, Spearman, and Pearson tests to assess the strength and direction of relationships between particle size *D*_*H*_ and the flow rate ratio (*Q*_*R*_). Kendall and Spearman tests evaluate monotonic trends without assuming a specific mathematical relationship. Pearson correlation, however, evaluates linear correlations and hence, *D*_*H*_ was also compared to $Q_{R}^{-1}$ and $Q_{R}^{-2}$ . All three tests were run using the “corr” function in Matlab 2024b.

#### Curve Comparison

To compare the size trends between formulations using 1 wt % phytantriol in pure ethanol or containing also 29 wt % water in the precursor solution, we used analysis of covariance (ANCOVA). This test determined if the two data sets were statistically different in their slopes or intercepts. The test was run using the “fitlm” function in Matlab 2024b.

#### Statistical Significance

For all statistical tests, a *p* value less than 0.05 was considered statistically significant.

## Results and Discussion

In this study, cubosomes are produced by solvent exchange. This method enables the assembly of phytantriol molecules into cubosomes through a nucleation and growth mechanism.^65^ As assembly proceeds, the F127 copolymer in the aqueous phase is expected to coat the forming particles, providing steric stabilization once surface coverage becomes high enough.^66^ The resulting dispersions are colloidally stable for ca. 1 week.

A key focus in this study is on controlling cubosome size by tuning the mixing conditions using a microfluidic hydrodynamic flow-focusing device. The phytantriol-ethanol solution is injected through the middle inlet (flow rate *Q*_*m*_) and is focused by two aqueous F127 streams flowing from the side channels (each at *Q*_*S*_). Mixing occurs across the two ethanol:water interfaces in the outlet channel, where ethanol and water rapidly interdiffuse. Because phytantriol monomers diffuse an order of magnitude slower, this abrupt change of solvent polarity initiates phytantriol self-assembly. The flow rate ratio *Q_R_=2Q_s_/Q*_*m*_ (eq 2) is a crucial parameter in this approach, controlling both the dilution rate and width of the focused stream (eq 3). Under these conditions, generally stable flow without visible precipitation of phytantriol is achieved using initial phytantriol in ethanol concentrations (*C*_*phyt.inint*_) up to 2 wt % and *Q*_*R*_ values up to 30, resulting in dilute cubosome dispersions. For consistency, all samples are diluted after formation to match the final composition of *Q*_*R*_=30 (Table S1).

### SAXS and Cryo-TEM Confirm Cubosome Formation

Before analyzing the effect of *Q*_*R*_ on cubosome size, it is important to verify their internal structure. Figure 2a shows SAXS patterns for two samples prepared at *Q*_*R*_ = 10 and 15. The former shows six Bragg peaks consistent with the *Pn*3̅*m* cubic phase; the latter shows only the first two, but at the same *q* positions, indicating the same internal structure and lattice parameter. This is expected since both compositions lie in the two-phase *Pn*3̅*m* with excess water region of the phytantriol-water phase diagram (Figure 1a),^18^ which begins at 28 wt % water.

The observed Bragg peaks were indexed as the {110}, {111}, {200}, {211}, {220}, and {221} reflections of the *Pn*3̅*m* space group. Plotting the measured *q_hkl_* values against (*h* ^2^+*k* ^2^+*l* ^2^)^1/2^ yields a linear fit passing through the origin (Figure 2b), from which the lattice parameter *a* = 6.7 nm is extracted.^67,68^ The bilayer thickness (*δ*) can be estimated through eq 7, yielding *δ* = 2.87 nm, which is consistent with reported values for the lamellar phase.^18^

Fitting the first Bragg peaks with a Lorentzian function (eq 8) provides a lower bound approximation of the domain size *ξ*, yielding ≳81 nm for *Q*_*R*_ = 10 and ≳65 nm for *Q*_*R*_ = 15. Since *ξ* is constrained by particle size, and all other experimental conditions are identical, this supports that higher *Q*_*R*_ produces smaller cubosomes.

At *q*∼0.045Å^–1^ a broad peak is observed, indicating loose periodicities with an average spacing of ca. 14 nm. It is unclear whether this could be attributed to loosely packed bilayers at the edges of cubosomes (as is observed in some cryo-TEM images – Figure 3) or to a less obvious feature. However, we rule out that this is a characteristic of the cubosome form factor, since the expected *qR* for the first minimum (∼3–4.5) would imply particle sizes far below those observed with DLS.

Figure 3 shows cryo-TEM images of samples prepared at *Q*_*R*_ of 8, 10, and 15. In this case, samples were concentrated to 2 wt % by centrifugation, using Amicon filters with 35 kDa membranes. The images unequivocally confirm the presence of cubosomes across all samples, alongside a significant number of vesicles. The presence of vesicles is a common feature on cubosomes samples, and often related to heterogeneities in particle composition. The stabilizer may affect the self-assembly of Phytantriol thereby producing dispersions of lamellar phases.^31,32,69^ In future work, this could potentially be mitigated by exploring alternative stabilizers.

Approximately 15 regions were imaged per sample, providing sufficient detail to confirm the presence and structural features of both species. Notably, many cubosomes are observed in the size range of approximately 60–80 nm, consistent with domain size measurements provided by SAXS (Figure 2c). Regarding the vesicles, while many display double-bilayer structures, a significant number are also encased in a darker, diffuse region ca. 7 nm thick (Figure 3). This coating fully envelops some vesicles and partially covers others. Although the exact nature of this coating is unclear, it is plausible that it could be related to the F127 coating.

While the number of imaged regions per sample is limited to yield statistically significant data for determining size distributions and number concentrations, an inspection of the images still suggests cubosome number fraction of 55%, 37%, and 65% for *Q*_*R*_ values of 8, 10, and 15, respectively, pointing to similar amounts of cubosomes and vesicles, and with comparable sizes.^6,26^ Despite their similar sizes, their compositions are markedly different. Cubosomes are expected to contain only 28 wt % water, compared to vesicles, which are mostly water. This suggests that the majority of the lipid content is contained within the cubosomes. For example, an estimated comparison between a 70 nm cubosome and a vesicles of the same size and bilayer thickness indicates that the cubosome would contain approximately 85% of all the lipid material.

Cubosomes are expected to dominate the signal in DLS measurements, being composed of ca. 72% of lipid, thus providing significantly higher contrast to the solvent than vesicles.

While cryo-TEM suggests cubosome sizes peaking around 50–60 nm, consistent with the domain sizes obtained by SAXS, these values are considerably smaller than the hydrodynamic diameters measured by DLS (Section 3.2). This discrepancy is expected, as DLS results are intensity-weighted, which emphasize larger particles and shift the apparent mean size upward. Simultaneously, cryo-TEM probes a limited number of sample regions, and larger particles may be excluded from the carbon grid. Therefore, due to the much larger number of particles sampled and the ability to rapidly acquire repeatable measurements, DLS is used as the primary method to quantitatively compare cubosome sizes across different *Q*_*R*_ values and precursor compositions.

### DLS Reveals Cubosome Size Dependence on *Q*_*R*_

Figure 4 presents the DLS results used to characterize cubosome size. Figure 4A shows representative autocorrelation functions at 90° and 173°, from which the diffusion coefficients and hydrodynamic diameter (*D_H_*) are obtained. In general, the curves can be suitably fit with the cumulants method, with good overlap among the triplicate measurements, indicating low-to-moderate polydispersity. Across microfluidic samples, the PDI interquartile range is 0.15–0.19, with only three outliers (PDI = 0.08, 0.026, and 0.028), indicating reasonably narrow size distributions. Bulk-prepared cubosomes also show generally moderate PDI (interquartile range of 0.17–0.21), but for *C_phyt.init_* = 2 wt %, a second population of micron-sized particles appears in some 173° curves (Figure S1).

Figures 4B,C show the average *D_H_* for cubosomes prepared using microfluidic (Figure 4B) or bulk (Figures 4C) solvent exchange. For simplicity, both are plotted as functions of *Q*_*R*_ and dilution ratio (*Dil_R_*). However, strictly speaking, *Q*_*R*_ applies only to microfluidic mixing. It is readily apparent that, overall, cubosomes prepared via hydrodynamic focusing in the microfluidic device tend to have smaller particles sizes as the *Q*_*R*_ increases. This trend is consistent across all *C_phyt.init_* values. While some variability in size is still significant—largely due to flow instabilities (pulsation, vibration, and use of a commercial device not specifically designed for this application)—the observed trends are statistically significant for all *C_phyt.init_*. Table 1 shows significant statistical correlations for each condition. Kedall’s τ (−0.54 to −0.60) and Spearman’s ρ (−0.70 to −0.72) give strong negative correlations for the effect of *Q*_*R*_ on *D_H_*, indicating monotonic decreasing size. Pearson’s *r* for the inverse relation *D_H_* vs 1/*Q_R_* shows a strong positive correlation (0.65 to 0.78), suggesting again decreasing sizes with *Q*_*R*_, and an approximately linear relationship with 1/*Q_R_*. Notably, Pearson’s *r* for *D_H_* vs $1/Q_{R}^{2}$ yields similarly strong correlations (0.66 to 0.79, see SI, Table S7), indicating that the trend could also be captured by an exponent of −2, or more generally, by a power-law dependence on *Q*_*R*_ with an exponent between −1 and −2. This is perhaps not surprising since the width of the focused stream *w_f_* is proportional to 1/*Q_R_* (eq 3), and the diffusion mixing time *τ_mix_* is proportional to $1/Q_{R}^{2}$ (eq 4). Indeed, the shape of the *τ_mix_* vs *Q*_*R*_ curve (Figure 4D) closely resembles the *D_H_* vs *Q*_*R*_ curves from microfluidics (see Figure 4B,D), suggesting that both *w_f_* and *τ_mix_* play key roles in determining *D_H_*. The p-value across these statistical tests is below 0.010 indicating statistical significance for the microfluidic samples (Table 1).

**Table 1:** Correlation Coefficients for Particle Size *D*_*H*_ and Flow Rate Ratio *Q*_*R*_ ^a^

		Kendall (*D*_*H*_ vs *Q*_*R*_)		Spearman (*D*_*H*_ vs *Q*_*R*_)		Pearson (*D*_*H*_ vs 1/*Q_R_*)	
*C*_hyt.EtOH_/wt %	Nr. Points	τ	*p*-value	ρ	*p*-value	*r*	*p*-value
Microfluidics							
1.0	3 × 5	–0.535	0.010	–0.698	0.004	0.782	<0.001
1.5	3 × 5	–0.712	<0.001	–0.854	<0.001	0.769	0.0013
2.0	5 × 5	–0.548	<0.001	–0.710	<0.001	0.701	<0.001
1.0 (29%w)	3 × 5	–0.727	<0.001	–0.853	<0.001	0.881	<0.001
Bulk							
1.0	2 × 5	0.072	0.855	0.124	0.734	–0.113	0.757
1.5	2 × 5	0.548	0.044	0.729	0.017	–0.655	0.040
2.0	2 × 5	0.283	0.315	0.369	0.294	–0.343	0.339
1.0 (29%w)	2 × 5	0.754	0.005	0.886	<0.001	–0.8537	0.002

a Kendall’s τ and Spearman’s ρ evaluate monotonic trends without assuming linearity. Pearson’s *r* coefficients are provided to evaluate the correlation and linearity of *D*_H_ vs $Q_{R}^{-1}$ .

In contrast, cubosomes prepared in bulk show markedly different behavior, with only a weak and generally nonsignificant trend toward larger particle sizes at higher dilution ratios. This increase is consistent with nanoprecipitation, where lower solute concentrations tend to result in lower supersaturation, fewer *nuclei*, and consequently more extensive particle growth.^65^ The weak trend in bulk can also be attributable to the uncontrolled mixing process. As the injected phytantriol-ethanol solution is stirred into the F127 solution, eddies of variable sizes form, leading to polydisperse mixing lengths and gradients in ethanol concentration.^65^ This polydispersity in mixing lengths results in variable solvent exchange rates, undermining control over supersaturation and particle size.

In the microfluidic system, on the other hand, mixing occurs at two well-defined interfaces, where the average focused stream width *w̅_f_* is controlled by *Q*_*R*_ (eq 3) and fluid viscosity. Although higher *Q*_*R*_ also implies greater dilution, the local phytantriol concentration in the focused stream remains nearly constant due to its much slower diffusion compared to the solvents. Hence, increasing *Q*_*R*_ accelerates solvent exchange (shorter *τ*_*mix*_) while maintaining the local phytantriol concentration during assembly, promoting supersaturation and nucleation rather than growth.

Hence, *Q*_*R*_ provides a tunable means to control mixing length and mixing time, influencing cubosome size in a more reproducible manner. We anticipate that a custom-designed microfluidic device incorporating design elements to minimize pulsation, vibration, and other external perturbations, could further reduce experimental variability.

These findings are broadly consistent with those of Pilkington et al., who also observed a decrease in cubosome size with increasing *Q*_*R*_ using a related microfluidic approach.^54^ While their data exhibits smaller error bars, their particle sizes are generally larger than those reported here for comparable *Q*_*R*_ values. This difference is discussed in greater detail in the following section.

### Influence of Mixing and Flow Conditions on Cubosome Size

Cubosome formation via solvent exchange involves multiple steps: solvent mixing, supersaturation, nucleation, growth, aggregation and stabilization. All these rates are influenced by the local solvent composition and concentrations, which are controlled by *Q*_*R*_ and the initial solvent compositions. Among these steps, nucleation is particularly critical, as higher nucleation rates yield more particles (*N*_*cub*_), and thus smaller sizes (since $D_{H}^{3}\propto N_{Cub}^{-1}$ ). The nucleation rate *J* scales as exp­{−[ln­(*S*)]^−2^} where the supersaturation $S≡\left(C_{phyt}/C_{phyt}^{*}\right)$ depends on the phytantriol concentration *C*_*phyt*_ and its solubility $C_{phyt}^{*}$ . In the focused stream, the local phytantriol concentration *C_phyt_* ≈ *C_phyt.init_* remains nearly constant due its slow diffusion relative to water and ethanol, while $C_{phyt}^{*}$ drops rapidly during solvent exchange. This drives an abrupt increase in *S* and the onset of nucleation. The time scale over which solvent mixing occurs, *i.e.*, the mixing time (*τ*_*mix*_), should have a profound effect on the nucleation and growth process, which in turn are key in determining particle size.

The balance between *τ*_*mix*_ and the time needed for phytantriol to self-assemble into cubosomes (τ_assbl_) can be expressed by the Damköhler number for precipitation:

13 $$Da_{p}=\tau_{mix}/\tau_{assbl.}$$

*Da*_*p*_ is intrinsically linked with the balance between nucleation and growth rates of cubosomes or their seeds, which in turn, will determine the size of the final cubosomes. Values of *Da*_*p*_ *<1* indicate that solvent mixing occurs faster than cubosome nucleation, hence leading to homogeneous supersaturation and favoring nucleation over particle growth. These conditions lead to the smaller limit on the size of particles. In contrast, *Da*_*p*_ *>1* indicates that assembly starts before ethanol and water are fully mixed, resulting in fewer *nuclei* and larger final particle sizes. These conditions lead to transport-controlled kinetics and to larger particle sizes.^40^ These observations are broadly consistent with the data in Figure 4, since for the three different concentrations, the cubosome size tends to decrease as the *Q*_*R*_ increases (and *τ*_*mix*_ and *Da*_*p*_ decrease), asymptotically reaching a lower size limit. This lower size limit may stem from two factors: first, due to *Da*_*p*_ approaching values smaller than one; second, due to the decreasing rate of *τ*_*mix*_ itself, as dictated by the inverse square dependence of *Q*_*R*_ on *τ*_*mix*_ (eqs 3 and 4 and Figure 4D). Given the strong dependence of the focused width on the channel width (eq 3), further narrowing the device geometry, for instance to 50 × 50 μm^2^ channels, could yield thinner focused streams, shorter *τ*_*mix*_ values, and potentially enable the formation of sub-100 nm cubosomes, closer to the limit allowed by *τ*_*assbl*_ .

Using eq 4 and *D*_*m*_ ≈ 6.5 × 10^–10^ m^2^/s,^59^ we estimate *τ*_*mix*_ to go from ∼21 to ∼2 ms, as *Q*_*R*_ increases from 8 to 30 (Figure 4D). Since *τ*_*mix*_ is much smaller than the average fluid residence time *τ*_*res*_ (∼0.5 s), the final particle size should be primarily determined by the number of *nuclei* formed, leading to a simplified model of:

14 $$D_{H}=\alpha_{1}\cdot \tau_{mix}^{\alpha_{2}}+D_{H.0}$$

where *α*_*1*_ is a proportionality constant (that implicitly contains *τ*_*assbl*._), *α*_*2*_ is the exponent and *D*_*H.0*_ accounts for the small particle size limit when *Da*_*p*_ *<1*. Replacing *τ*_*mix*_ with *Q*_*R*_, through manipulation of eqs 3 and 4 leads to

15 $$D_{H}=\beta_{1}\cdot {\left(1+Q_{R}\right)}^{-\beta_{2}}+D_{H.0}$$

where the proportionality constant *β*_*1*_ implicitly contains *α*_*1*_, as well as *D*_*m*_ and *w*_*c*_, and *β_2_=2α*_*2*_ . eq 15 is consistent with the empiric observation that *D*_*H*_ decreases with *Q*_*R*_, and is supported by the strong Pearson correlations with $Q_{R}^{-1}$ and $Q_{R}^{-2}$ (Tables 1 and S7). Fits to the data (Figure 4B) yield *R* ^2^ values of 0.91–0.95 (Table 2). However, the fitted parameters, especially β_1_ and β_2_, carry substantial uncertainty due to the variability in the experimental data and the interdependence inherent in three-parameter models (*e.g.*, *β*_*1*_ and *D*_*H.0*_ can compensate for variations in *β*_*2*_). Supporting this, fixing *β*_*2*_ =2 produces equally reasonable visual fits, with just slightly lower *R* ^2^ values. Hence, the absolute parameter values in Table 2 should be interpreted with caution. It remains unclear whether the differences in the fitted exponents *β*_*2*_ (ranging from −2.6 to −1.9) result from experimental variability or reflect additional dependencies of size on *Q*_*R*_ or other factors (*e.g.,* concentration), not fully captured by this simplified model. Addressing this would require a more detailed study beyond the scope of the present work. Yet, the overall agreement with the observed experimental trends supports the plausibility of the model.

**Table 2:** Fitting Parameters for Proposed Model (eq 15)­^a^

*C_phyt.init_*/wt %	β_1_/10^–3^ arb. u.	β_2_	*D*_H.0_/nm	*R* ^2^	*β*_1_/10^–3^arb. u.	*D*_H.0_/nm	*R* ^2^
					*β*_2_=2		
1	1 ± 4	1.9 ± 1.6	133 ± 7	0.91	2 ± 5	134 ± 6	0.91
1.5	11± 21	2.5 ± 0.9	135 ± 5	0.98	4± 7	132 ± 8	0.97
2	3 ± 6	1.9 ± 1.2	160 ± 9	0.95	3 ± 7	160 ± 8	0.95
1 (29% water)	0.8 ± 1.7	1.3 ± 1.1	116 ± 20	0.94	3 ± 10	124 ± 11	0.93

a Values are reported as estimate ± standard error.

Regarding the results from Pilkington et al.,^54^ as mentioned above, cubosome sizes also decrease with increasing *Q*_*R*_, confirming the general trend observed in the present work. However, for comparable *Q*_*R*_ values (10–30), their sizes (ca. 225–190 nm at 0.59 wt % phytantriol) are significantly larger than those observed here (ca. 148–134 nm at 1 wt %). While some compositional parameters are different (they fix the lipid-to-F127 molar ratio at 10, whereas we fix the F127 concentration at 0.1 wt %), this alone is unlikely to explain the difference, as higher F127 amounts typically favor smaller sizes. More likely, the differences arise from the device geometry and flow conditions. Their larger channel cross-section (150 × 150 μm^2^ vs our 100 × 100 μm^2^) results in wider focused streams *w*_*f*_ and ∼2.25x longer *τ*_*mix*_ (eqs 3 and 4), meaning that a *Q*_*R*_ of 30 in their system may correspond to ∼20 in ours. In addition, their higher total flow rates (400–480 μL/min) and shorter outlet channel (35 mm) leads to a residence time *τ*_*res*_ of ∼0.1s. For *Q*_*R*_ = 10, this corresponds to a *τ_res_/τ*_*mix*_ ratio of ∼3, compared to ∼34 in our system, indicating that mixing in our system is more complete before the fluid exits the device. While the low error bars and PDIs they report suggest that assembly remains well controlled, the higher *τ_res_/τ*_*mix*_ in our device may contribute to the consistently smaller particle sizes observed.^70^

### Cubosome Size Increases with Phytantriol Concentration

Another trend, not yet discussed, is the systematic increase in *D_H_* as the phytantriol concentration in the precursor solution *C_phyt.init_* increases. This is evident both in Figure 4B and in the fitted *D_H_*._0_ values, which rise from 133 nm at 1 wt % phytantriol to 160 nm at 2 wt %. At first glance, this contradicts the expectation that higher supersaturation yields more *nuclei* and smaller particles. However, the higher concentration should also induce supersaturation at lower water content in the central stream, thus reducing the cubosome assembly time *τ_assbl._*. Since the mixing time *τ_min_* remains largely constant for each *Q_R_*, a shorter *τ_assbl._* shifts the balance toward growth over nucleation, leading to larger particles. This is consistent with an increase in the Damköhler number *Da_p_* (eq 13), which is associated with larger particle sizes.^57^

A complementary explanation involves the constant stabilizer (F127) concentration used throughout the study. As solvent exchange occurs rapidly in the focused stream, F127 is expected to diffuse into this region more slowly, encountering cubosomes or their precursors already forming. During this process, F127 adsorbs onto these *nuclei*, helping to minimize aggregation. While the overall amount of F127 is suitable in bulk solution (given the low concentration of phytantriol), the situation may differ in the microfluidic device. Locally, F127 must diffuse into the focused stream, and increasing the lipid concentration reduces the local F127:lipid ratio. This could result in insufficient coverage of forming cubosome seeds, potentially increasing the likelihood of early stage aggregation and contributing to the observed systematic increase in particle size at higher *C_phyt.init_*. Yet this early stage aggregation, particularly under conditions where some ethanol remains (as at low *Q_R_* before the dilution step to *Dil_R_* of 30), could plausibly reflect a controlled fusion process, similar to the formation of lipid nanoparticles that fuse until the surface becomes saturated with PEG-based stabilizers.^71^ If this hypothesis is confirmed, the time scale of F127 coverage of *nuclei* would also need to be considered, alongside solvent mixing (*τ_min_*) and particle assembly (*τ_assbl._*), in determining final particle size.^65^

Distinguishing whether the observed size increase with phytantriol concentration is primarily driven by a higher *Da_p_* or by a lower local F127:lipid ratio would require further dedicated studies and is beyond the scope of the present work. Nonetheless, despite the lack of a complete mechanistic understanding, our empiric model is consistent with a prominent role of *τ_min_* and its dependence on *Q_R_*. Importantly, by tuning *Q_R_* and the initial lipid concentration, we achieve control over cubosome sizes ranging from 195 down to 135 nm.

### Impact of Water Inclusion in the Precursor Solution on Cubosome Size

We have seen in the previous sections that supersaturation is critical for cubosome formation. Given the asymptotic behavior of both the focused stream width *w_f_* and mixing time *τ_min_* with *Q_R_*, further increasing *Q_R_* beyond 30 results in progressively less significant changes in these parameters. Additionally, further increasing *Q_R_* leads to dilution, which eventually becomes impractical. To further increase nucleation rates and decrease particle sizes while staying within the same *Q_R_* range, we hypothesized that supersaturation could be reached more rapidly if some water was included in the precursor solution. While the rate of mixing is still governed by *Q_R_* (via *w_f_*), the time to reach the nucleation threshold would be reduced if some water is already present from the beginning in the focused stream. This could increase nucleation and reduce particle size by lowering *Da_p_*. We tested this using a 29 wt % water precursor (phytantriol/ethanol/water of 1/70/29 wt %). The solution is stable and suitable for injection in the microfluidic device, but close to precipitation by further addition of water. Higher phytantriol concentrations under these conditions were not stable enough. The results are shown in Figure 5, with the best-fit parameters included in Table 2.

**Figure 5:**
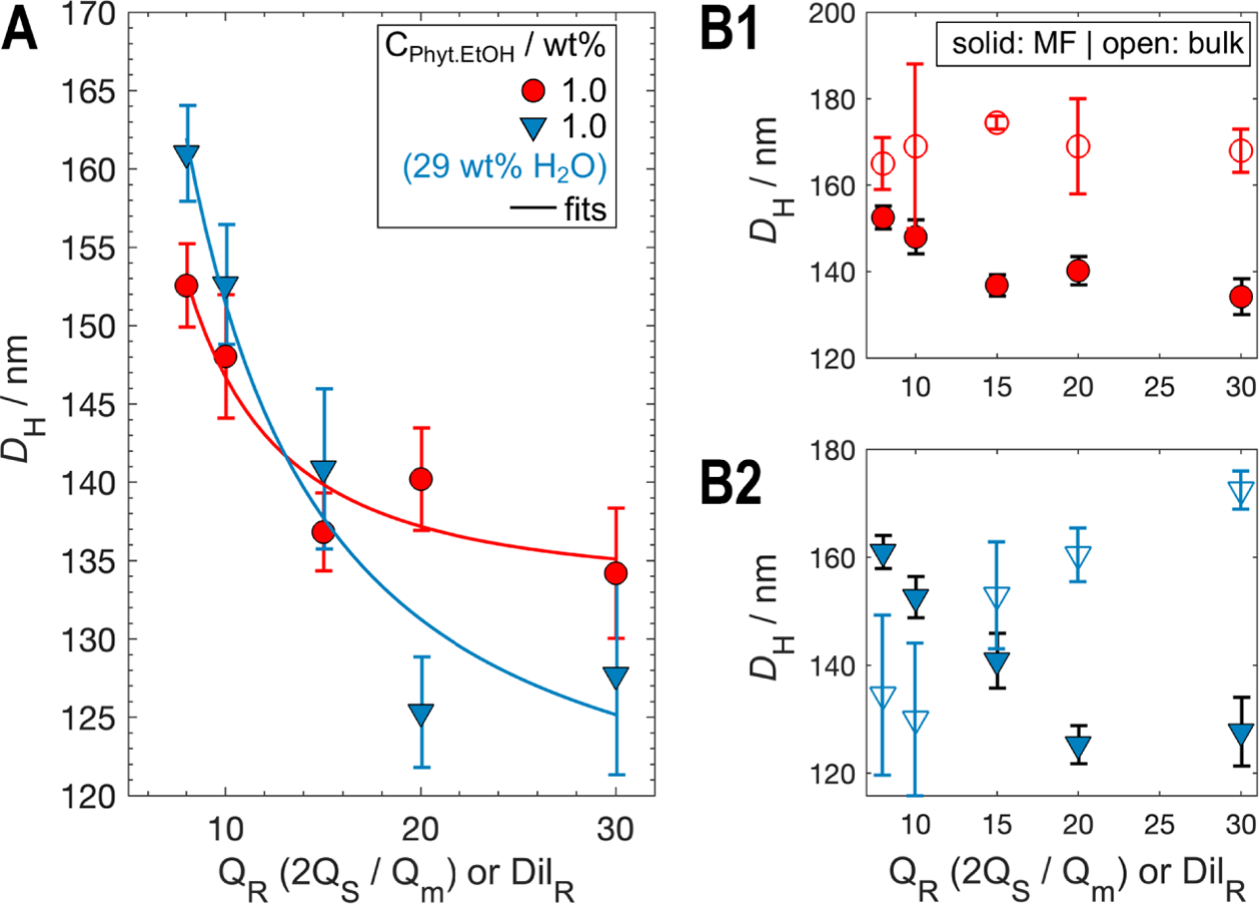
Influence of water in the precursor solution. (A) The trend of decreasing D_H_ with Q_R_ is preserved when 29 wt % water is added to the 1 wt % phytantriol precursor solution. However, the slopes of the D_H_ vs Q_R_ curves for the ethanol-only (red circles) and ethanol–water (blue downward triangles) precursors differ slightly. When plotted as D_H_ vs $Q_{R}^{-1}$ (not shown), both the slopes and intercepts differ with statistical significance (*p* = 0.01). (B) Comparison between microfluidic samples (filled symbols) and their bulk counterparts (open symbols). B1 shows the results for ethanol-only precursor solutions, while B2 shows the results for precursor solutions containing 29 wt % water. The stabilizer concentration was constant across all samples (0.1 wt % F127).

As with the previous microfluidic samples, also here we observe a strong decrease in *D*_*H*_ with increasing *Q*_*R*_, as confirmed visually and by Kendall, Spearman and Pearson tests (Table 1). This is in contrast with bulk preparation, where a statistically significant increase in *D*_*H*_ with increasing *Q_R_* is observed. However, a more nuanced pattern emerges when comparing precursor formulations in the microfluidic assembly. At high *Q_R_*, samples prepared with 29 wt % water in the precursor yield smaller *D*_*H*_ values than those with pure ethanol, as hypothesized. At low *Q_R_*, however, the trend reverses, with the ethanol–water precursor producing larger particles.

While both data sets have sizable error bars, we assessed the significance of these differences using ANCOVA on *D*_*H*_ vs $Q_{R}^{-1}$ . The analysis revealed statistically significant differences in both slope (*p* = 0.010) and intercept (*p =* 0.013) when comparing the two formulations, thus suggesting that they have distinct linear relationships between *D*_*H*_ and $Q_{R}^{-1}$ . Similar results were also found for *D*_*H*_ vs $Q_{R}^{-2}$ (slope: *p* = 0.013; intercept: *p* = 0.030).

Fitting the data with the empirical model (eq 15 and Figure 5) provides once again a reasonable fit to the data (*R* ^2^ = 0.94, Table 2). When fitting without constraints (except for a maximum *D_H.0_* of 140 nm), we obtain *β*_2_ = 1.3 ± 1.1, which is somewhat different from the case in pure ethanol. Still, this uncertainty is high, and these absolute values should be taken with caution (for instance, fixing *β*_2_ to 2 would still provide a reasonable *R* ^2^ value of 0.93, highlighting this uncertainty). Altogether, the observation that *D*_*H*_ is larger for samples with 29 wt % water than for those with pure ethanol at low *Q_R_* (contrary to the expectation that added water would increase nucleation and reduce sizes), and smaller at high *Q_R_* (consistent with the hypothesis), appears robust.

The apparent contradiction between the hypothesis and the results could be explained by considering, again, the intricate interplay of F127 transport and its role in cubosome stabilization. At low *Q_R_* the focused stream is wide, so F127 must diffuse further to reach nucleating cubosomes. If nucleation is indeed faster in the presence of water, *nuclei* formed early at low *Q_R_* may remain with lower F127 coverage for longer periods of time, increasing the likelihood of fusion or aggregation. At high *Q_R_*, where the focused stream is narrow, F127 reaches nuclei more rapidly, allowing these faster forming *nuclei* to be stabilized rather quickly and resulting in smaller final particle sizes. As noted in the previous section, this aggregation at lower *Q_R_*, in the presence of some ethanol, could involve fusion processes similar to those seen in lipid nanoparticle formation, where fusion proceeds until surfaces are saturated with PEG-based stabilizers.^71^

Overall, while the underlying mechanisms are complex, the inclusion of water in the precursor provides an additional strategy for tuning cubosome size, allowing particles as small as ∼ 125 nm to be achieved within the same *Q_R_* range. Our empirical model (eq 15) accommodates these complex trends phenomenologically across varying precursor compositions and flow conditions. This efficacy is likely due to the central role of *Q_R_* (coupled with the three fitting parameters) in controlling the flow and mixing in the device. *Q_R_* controls solvent exchange rates, driving nucleation and initial growth, thus having a strong influence in particle size. In parallel, it also governs the transport of the F127 stabilizer, which may be important in defining regimes where controlled aggregation or fusion may occur, particularly at low *Q_R_*.

## Conclusions

We have shown that a hydrodynamic flow-focusing microfluidic approach for solvent exchange enables controlled assembly of cubosomes with tunable sizes ranging from ∼195 nm down to ∼125 nm. The size is strongly influenced by the flow rate ratio *Q_R_*, which defines the width of the focused precursor stream and, consequently, the solvent mixing time and stabilizer transport. This, in turn, affects the kinetics of nucleation, growth, and nuclei aggregation during cubosome self-assembly, culminating in cubosomes with tunable size. SAXS and cryo-TEM confirm the internal *Pn*3̅*m* cubic phase, and DLS measurements show a statistically significant reduction in cubosome size with increasing *Q_R_*. This is in stark contrast with the weak and generally nonsignificant trend toward larger particle sizes observed with bulk mixing.

A simple model based on the Damköhler number (*Da*_*p*_ = *τ_min_*/*τ_assbl._*) provides a useful framework for interpreting the interplay between solvent exchange dynamics and lipid assembly kinetics, and their influence on controlling size. Addition of water to the precursor stream further expands the tunability window by allowing smaller sizes at high *Q_R_* values.

While the resulting dispersions display a low-to-moderate polydispersity, a significant fraction of vesicles remains, as often observed in F127-stabilized systems. This could potentially be mitigated through optimization of formulation parameters, such as alternative stabilizers or buffer composition.

Despite some batch-to-batch variability—largely due to flow instabilities and limitations of the commercial device—the observed trends are statistically significant across all tested conditions with microfluidics. A dedicated device engineered to reduce flow and external instabilities could further improve consistency.

Given the importance of size control in drug and gene delivery applications, this approach represents a promising alternative over traditional top-down and bulk solvent-exchange methods. Future work should aim, on one hand, to reduce vesicle formation and improve scalability, while on the other expand to elucidate better the interplay between lipid and stabilizer concentration with *Q_R_*. In addition, exploring narrower channel geometries (*e.g.*, 50 × 50 μm^2^) could further shorten mixing times and potentially extend the tunability window to sub-100 nm cubosomes.

## Supplementary Material



## Data Availability

All data and Matlab scripts used for the analysis and for generating the figures and tables are publicly available on Zenodo, DOI: 10.5281/zenodo.15794568

## Notes

Table S1: microfluidic flow rates and volumes used for all starting lipid concentrations. Table S2: stock solution volumes used for bulk cubosome assembly. Tables S3–S5: DLS results for cubosomes assembled from 1.0, 1.5, and 2.0 wt % phytantriol in ethanol precursor solutions. Table S6: DLS results for cubosomes assembled from 1/70/29 wt % phytantriol/ethanol/water precursor solution. Table S7: Pearson correlation coefficients for *D_H_* vs $Q_{R}^{-2}$ Figure S1: DLS data showing two cubosome populations in selected bulk samples at 2.0 wt % phytantriol in ethanol precursor solution (PDF)
