# Supplementary material for: Microfluidic Flow-Focusing for Size-Controlled Formation of Cubosomes
Source: Langmuir. 2025 Oct 17;41(42):28434–46. doi: 10.1021/acs.langmuir.5c03488 (PMC12573805; doi:10.1021/acs.langmuir.5c03488)
Supplement: Supplementary file 1 [file la5c03488_si_001.pdf]

Supporting Information

Microfluidic Flow-Focusing for Size-Controlled Formation of Cubosomes

Celso J.O. Ferreira[a,b,c,d], Margarida Barros[a,b], Marco Fornasier[d], Alexandre Chícharo[a], Claudia Botelho[c], Maria Elisabete C.D. Real Oliveira[b], Ulf Olsson[d], Bruno F.B. Silva[a,e,f,g,*]

[a]INL-International Iberian Nanotechnology Laboratory, 4775-330 Braga, Portugal
[b]CF-UM-UP Physics Centre, Minho University, 4710-057 Braga, Portugal
[c]CEB-Centre of Biological Engineering, Minho University, 4710-057 Braga, Portugal
[d]Division of Physical Chemistry, Lund University, P.O. Box 124, SE-221 00 Lund, Sweden
[e]Empa, Swiss Federal Laboratories for Materials Science and Technology, Center for X-Ray Analytics, Lerchenfeldstrasse 5, 9014 St. Gallen, Switzerland
[f]Empa, Swiss Federal Laboratories for Materials Science and Technology, Laboratory for Biointerfaces, Lerchenfeldstrasse 5, 9014 St. Gallen, Switzerland
[g]Empa, Swiss Federal Laboratories for Materials Science and Technology, Laboratory for Biomimetic Membranes and Textiles, Lerchenfeldstrasse 5, 9014 St. Gallen, Switzerland
*Correspondence: bruno.silva@empa.ch

Number of pages: 7

Number of figures: 1

Number of tables: 7

Table of Contents

Table S1: Microfluidic flow rates and volumes used for all starting lipid concentrations. S2

Table S2: Stock solution volumes used in cubosome assembly by mixing in bulk. S2

Table S3: DLS results for cubosome samples assembled from 1 wt% phytantriol in pure ethanol precursor solutions. S3

Table S4: DLS results for cubosome samples assembled from 1.5 wt% phytantriol in pure ethanol precursor solutions. S4

Table S5: DLS results for cubosome samples assembled from 2 wt% phytantriol in pure ethanol precursor solutions. S5

Table S6: DLS results for cubosome samples assembled from phytantriol/ethanol/water (1/70/29 wt%) precursor solutions. S6

Table S7: *Pearson r for* D_H *vs.* Q_R^{-2} S6

Figure S1: Two cubosome populations are observed in some bulk-mixing samples at 2 wt% phytantriol in ethanol precursor solution. S7

***Table S1**: Microfluidic flow rates and volumes used for all starting lipid concentrations. $2Q_S$ is the combined flow rate of the F127 solutions from both side channel inlets. Q_m is the flow rate of the ethanol-phytantriol solution in the middle inlet. $V_{Collected}$ is the sample volume collected from the microfluidic device, which is then mixed at the outlet with an additional volume ($V_{F127.Added}$) of F127 solution to achieve a standardized final lipid concentration equivalent to a dilution ratio (Dil_R) of 30. The total final volume is 600 μL per sample.*

Q_R	$2Q_S$ / µL min^{-1}	Q_m / µL min^{-1}	$V_{Collected}$ / µL	$V_{F127.Added}$ / µL
8	88.89	11.11	174	426
10	90.91	9.09	213	387
15	93.75	6.25	310	390
20	95.24	4.76	406	194
30	96.77	3.23	600	0

***Table S2**: Stock solution volumes used in cubosome assembly by mixing in bulk. $V_{F127-aq.}$ and $V_{Lipid-EtOH}$ are the initial volumes of F127 aqueous solution and lipid-ethanol solution, respectively, used to produce the initial cubosome solutions in step 1. These volumes reproduce the same compositions obtained in the microfluidic device. From this initial mixture, a volume $V_{Step1.sol.}$ is taken and combined with an additional F127 solution, with volume $V_{F127.Added}$, to bring the samples to a common Dil_R of 30.*

	Step 1, mimicking microfluidic mixing for different Q_R		Step 2, dilution to constant concentration, equivalent to Dil_R=30	
Dil_R	$V_{F127-aq.}$ / µL	$V_{Lipid-EtOH}$ / µL	$V_{Step1.sol.}$ / µL	$V_{F127.Added}$ / µL
8	444	56	145	355
10	455	45	177	323
15	469	31	258	242
20	476	24	339	161
30	484	16	500	0

Table S3*: DLS results for cubosome samples assembled from 1 wt% phytantriol in pure ethanol precursor solutions. Parameters were obtained by fitting the autocorrelation curves with equations 8-11. Run 3 of the microfluidics condition was excluded from analysis due to irregularities in the autocorrelation curves, likely caused by a transient issue with the tubing.*

	Microfluidics			Bulk		
Run Nr.	Q_R	D_H / nm	PDI	Dil_R	D_H / nm	PDI
1	8	157.8	0.16	8	171	0.18
	10	152.6	0.18	10	150	0.18
	15	139.7	0.20	15	173	0.17
	20	134.4	0.18	20	158	0.19
	30	128.5	0.16	30	163	0.23
2	8	150.7	0.18	8	159	0.17
	10	151.3	0.16	10	188	0.16
	15	138.9	0.23	15	176	0.21
	20	145.7	0.16	20	180	0.17
	30	131.8	0.20	30	173	0.18
4	8	149.2	0.16			
	10	140.2	0.16			
	15	131.9	0.15			
	20	140.5	0.10			
	30	142.3	0.17			

***Table S4**: DLS results for cubosome samples assembled from 1.5 wt% phytantriol in pure ethanol precursor solutions. Parameters were obtained by fitting the autocorrelation curves with equations 8-11. Values marked with an asterisk (at Q_R =20, from microfluidic run 1) were excluded from analysis since they were acquired under conditions that differed from the rest of the dataset: either measured only at 173° (134.7 nm) or measured five days after sample preparation (180.4 nm).*

	Microfluidics			Bulk		
Run Nr.	Q_R	D_H / nm	PDI	Dil_R	D_H / nm	PDI
1	8	206.6	0.22	8	150	0.18
	10	189.0	0.21	10	150	0.23
	15	148.9	0.26	15	157	0.18
	20 (only 173°)	134.7*	0.17	20	163	0.24
	20 (after 5 days)	180.4*	0.28			
	30	139.9	0.13	30	192	0.20
2	8	159.2	0.20	8	162	0.15
	10	152.2	0.18	10	161	0.15
	15	137.9	0.19	15	176	0.18
	20	140.3	0.19	20	170	0.19
	30	142.6	0.16	30	165	0.17
3	8	170.9	0.11			
	10	154.9	0.19			
	15	144.3	0.17			
	20	135.0	0.18			
	30	136.4	0.19			

Table S5: *DLS results for cubosome samples assembled from 2 wt% phytantriol in pure ethanol precursor solutions. Parameters were obtained by fitting the autocorrelation curves with equations 8-11.*

	Microfluidics			Bulk		
Run Nr.	Q_R	D_H / nm	PDI	Dil_R	D_H / nm	PDI
1	8	190.2	0.13	8	154.2	0.16
	10	187.9	0.14	10	167.6	0.16
	15	161.4	0.15	15	161.3	0.18
	20	152.4	0.17	20	167.3	0.18
	30	137.3	0.23	30	190.4	0.24
2	8	194.3	0.19	8	205	0.19
	10	184.8	0.15	10	199	0.18
	15	168.4	0.14	15	208	0.21
	20	184	0.19	20	221	0.21
	30	182.3	0.17	30	218	0.25
3	8	205.9	0.12			
	10	198.2	0.11			
	15	185.5	0.13			
	20	169.3	0.15			
	30	187.5	0.11			
4	8	194.1	0.084			
	10	204.7	0.10			
	15	168.8	0.16			
	20	173.7	0.15			
	30	152	0.19			
5	8	192.4	0.12			
	10	171.8	0.17			
	15	158.4	0.16			
	20	151.9	0.18			
	30	165.1	0.15			

***Table S6**: DLS results for cubosome samples assembled from phytantriol/ethanol/water (1/70/29 wt%) precursor solutions. Parameters were obtained by fitting the autocorrelation curves with equations 8-11.*

	Microfluidics			Bulk		
Run Nr.	Q_R	D_H / nm	PDI	Dil_R	D_H / nm	PDI
1	8	167	0.10	8	145	0.16
	10	147	0.11	10	140	0.12
	15	151	0.12	15	146	0.17
	20	132	0.18	20	157	0.18
	30	140.1	0.20	30	175	0.18
2	8	157	0.13	8	124	0.18
	10	159.9	0.08	10	120	0.17
	15	137	0.18	15	160	0.16
	20	124	0.16	20	164	0.16
	30	119	0.21	30	170	0.16
3	8	159	0.10			
	10	151	0.09			
	15	134.6	0.14			
	20	120	0.21			
	30	124	0.18			

***Table S7:** Pearson r for D_H vs. Q_R^{-2}*

		microfluidics		bulk	
$C_{Phyt.EtOH}$ / wt%	Nr. Points	*r*	p-value	*r*	p-value
1.0	3x5	0.785	<0.001	-0.145	0.689
1.5	3x5	0.786	<0.001	-0.615	0.059
2.0	5x5	0.706	<0.001	-0.317	0.373
1.0 (29%w)	3x5	0.872	<0.001	-0.807	0.0048

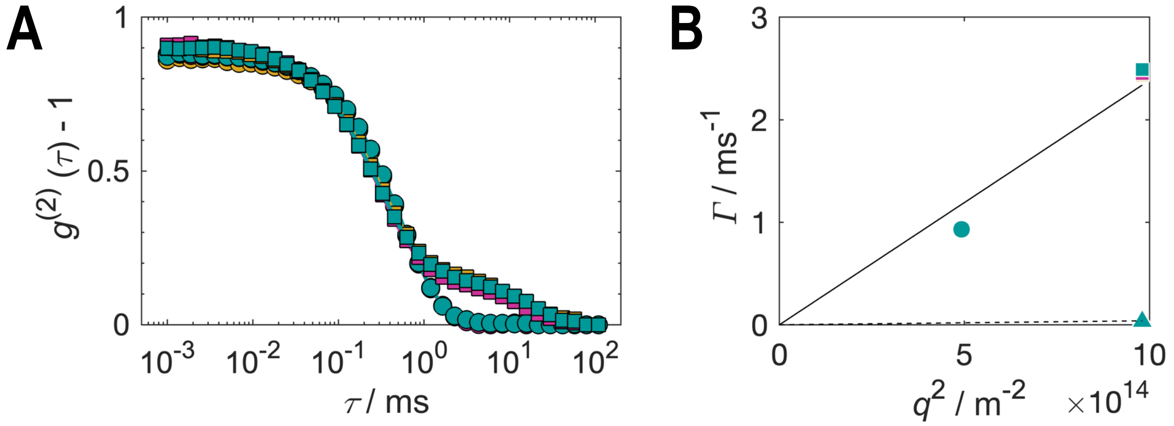

***Figure S1**: Two cubosome populations are observed in some bulk-mixing samples at 2 wt% phytantriol in ethanol precursor solution. Shown here is a sample at Dil_R=8 **A**, Autocorrelation functions $g^{(2)}(\tau)-1$ at two scattering angles (90°, circles; 173°, squares), with three repeats per angle. The second population is clearly visible at 173°. In such cases, the autocorrelation curve is fitted with a biexponential decay model to isolate the decay rates Γ from both populations. **B**, Corresponding decay rates Γ vs. q^2, used to extract the hydrodynamic diameter D_H (Eqs. 9 and 11) from six points (three per angle) plus the intercept. Because at 173° (q^2=9.8x10^{14} m^{-2}) each population has a distinct Γ, the fast diffusion Γ (squares) are plotted with the 90° Γ to obtain the size of the small population (straight line fit), while the slow diffusion Γ (triangles) are used to obtain a rough estimate (dashed line) of the large particle size (~11-17 μm).*
